# A Pilot Study of Adolescents with Psychotic Experiences: Potential Cerebellar Circuitry Disruption Early Along the Psychosis Spectrum

**DOI:** 10.1007/s12311-023-01579-5

**Published:** 2023-06-23

**Authors:** Caoimhe Gaughan, Anurag Nasa, Elena Roman, Dearbhla Cullinane, Linda Kelly, Sahar Riaz, Conan Brady, Ciaran Browne, Vitallia Sooknarine, Olivia Mosley, Ahmad Almulla, Assael Alsehli, Allison Kelliher, Cian Murphy, Erik O’Hanlon, Mary Cannon, Darren William Roddy

**Affiliations:** Department of Psychiatry, Royal College of Surgeons in Ireland, Education and Research Centre, Beaumont Hospital, Dublin 9, Ireland

**Keywords:** Cerebellar peduncles, Psychotic experiences, Adolescents, Superior cerebellar peduncle, Diffusion imaging

## Abstract

A berrant connectivity in the cerebellum has been found in psychotic conditions such as schizophrenia corresponding with cognitive and motor deficits found in these conditions. Diffusion differences in the superior cerebellar peduncles, the white matter connecting the cerebellar circuitry to the rest of the brain, have also been found in schizophrenia and high-risk states. However, white matter diffusivity in the peduncles in individuals with sub-threshold psychotic experiences (PEs) but not reaching the threshold for a definitive diagnosis remains unstudied. This study investigates the cerebellar peduncles in adolescents with PEs but no formal psychiatric diagnosis.

Sixteen adolescents with PEs and 17 age-matched controls recruited from schools underwent High-Angular-Resolution-Diffusion neuroimaging. Following constrained spherical deconvolution whole-brain tractography, the superior, inferior and middle peduncles were isolated and virtually dissected out using ExploreDTI. Differences for macroscopic and microscopic tract metrics were calculated using one-way between-group analyses of covariance controlling for age, sex and estimated Total Intracranial Volume (eTIV). Multiple comparisons were corrected using Bonferroni correction.

A decrease in fractional anisotropy was identified in the right (*p* = 0.045) and left (*p* = 0.058) superior cerebellar peduncle; however, this did not survive strict Bonferroni multiple comparison correction. There were no differences in volumes or other diffusion metrics in either the middle or inferior peduncles.

Our trend level changes in the superior cerebellar peduncle in a non-clinical sample exhibiting psychotic experiences complement similar but more profound changes previously found in ultra-high-risk individuals and those with psychotic disorders. This suggests that superior cerebellar peduncle circuitry perturbations may occur early along in the psychosis spectrum.

## Introduction

Recent studies have redefined the phenotype of psychosis based on a spectrum rather than distinct diagnostic entities [1]. This continuum takes into consideration the variable severity of psychotic experiences (PEs) and the staged thresholds of certain conditions (for example initial attenuated psychotic symptoms can progress to a diagnosis of schizophrenia). A systematic review found that among 19 population studies, the median prevalence of psychotic experiences was 17% among children aged 9–12 years and 7.5% among adolescents aged 13–18 years [2]. Despite this evidence of frequent hallucinations and delusions in adolescents, only a small portion of the population will meet the threshold criteria of a diagnosable psychotic disorder such as schizophrenia. The development of this phenotype suggests that a state exists in which an individual may experience sub-threshold PEs in the absence of a disorder [3]. At the other end of the continuum lies more severe, debilitating psychiatric conditions such as schizophrenia. Although many who experience psychotic symptoms do not develop diagnosed disorders, there has been great evidence of the increased risk of clinical diagnosis later in life within these populations [4, 5]. As such, the identification of biological correlates may be important in the diagnosis and identification of high-risk populations to reduce the risk of poor mental health outcomes including suicidal behavior [6], poor socio-occupational function [7, 8], and neurocognitive deficits [9, 10].

The cerebellum has traditionally been viewed in light of its motor functions with evidence in both animal and human studies of cerebellar damage causing of ataxia and other movement disorders [11, 12]. Traditional cerebellar motor functions include ocular control, timing and voluntary limb movements, and sensorimotor coordination [13-15]. More recently, functional magnetic resonance imaging (fMRI) studies have been used to identify regions of the cerebellum associated with these functions [16] along with a greater appreciation of the cognitive and emotional functions of the cerebellum [17-19]. Andreasen’s theory of cognitive dysmetria lays the foundations for the investigation of the role of the cerebellum in psychosis and suggests that a lack of coordination within the cortico-thalamic-cerebellar network is associated with cognitive abnormalities in people with schizophrenia [20]. Decreased cerebellar expression of reelin mRNA and protein, needed for proper neural migration, has been found in postmortem studies of patients with psychosis [21, 22], in particular in the cerebellar Purkinje cells [23]. Reduced Purkinje neuron size has been found in the the anterior lobe and vermis of the cerebellum in postmortem studies [23, 24]. Cerebellar kynurenic acid, a kynurenine pathway metabolite thought to be protective against excitotoxicity [25], has also been found to be inversely correlated with previous psychosis scores in post mortem patients in schizophrenia [26]. Investigations of the cerebellar cognitive affective syndrome (CCAS) have also found lesions in the posterior lobe and vermis of the cerebellum associated with executive function impairments, including working memory, planning and verbal fluency, as well as blunted affect [27]. CCAS also incorporates psychiatric symptoms including mood and personality dysregulation, obsessive–compulsive tendencies, and psychotic experiences [27]. Due to the complex and polysynaptic nature of cerebellar and cerebellocortical circuitry, it has historically been difficult to identify whether these cerebellar areas can also be mapped to non-motor cortical regions [17].fMRI data of cerebellar cortical functional topography suggests that motor processing occurs within lobules I to VI and lobule VIII, with attention/executive and default-mode processes occurring in lobules VI to crus I, and crus III to VIIB [28]. Emotion processing is thought to be centrally located with a tendency to involve the cerebellar vermis [29]. Vestibular functions are also associated with vermal activation, namely in lobules V to VII, as well as lobules IX and X [28]. As such, there is overlap between oculomotor control and attention, as well as vestibular and emotional processing. Language processing is right lateralized, mirroring the left lateralization of cerebral cortical language processing [30]. In schizophrenia, a recent functional connectivity study found that the principal motor-to-supramodal gradient is compressed due to increased gradient values in sensorimotor regions and decreased gradient values in supramodal regions [31]. Additionally, hyperconnectivity between cerebellar sensorimotor and cognition areas, cerebellar cognition and cerebral sensorimotor areas, and vice versa has also been uncovered [31].

The cerebellar peduncles contain the principal efferent and afferent tracts of the cerebellum, connecting the deep cerebellar nuclei and parts of the cerebellar cortex to other parts of the central nervous system. The middle cerebellar peduncle (MCP) exclusively carries fibers from the contralateral pontine nuclei conveying signals from the cerebral cortex via the pontocerebellar tract [32]. Meanwhile, the inferior cerebellar peduncle (ICP) carries the afferent olivocerebellar, vestibulocerebellar, posterior spinocerebellar, rostral spinocerebellar, reticulocerebellar and trigeminocerebellar tracts, and efferent fibers via the cerebellovestibular tract [32]. The superior cerebellar peduncle (SCP) carries the afferent anterior spinocerebellar tract, and the efferent cerebellorubral, cerebellothalamic and nucleoolivary tracts [32]. The SCP carries projections to the dentate nucleus of the cerebellum, thought to be involved in cognition and perturbed in individuals at high risk for psychosis [33]. Connections between the cerebellum and hypothalamus also pass through the SCP, allowing cerebellar control of non-cognitive functions including hunger, sex drive, autonomic control and emotional/stress responses [34, 35].

Cerebellar connectivity is altered in people with schizophrenia or other psychotic disturbances, including those at high risk of psychosis. Reduced functional connectivity between the cerebellar and the left pre-supplementary motor area (preSMA), right anterior prefrontal cortex (aPFC) and precuneus is seen in first episode psychosis [36]. Similarly, reduced functional connectivity between the right supplementary motor area (SMA), left parahippocampal gyrus (PHG), right PHG and right superior temporal gyrus is seen in early schizophrenia and clinically high-risk groups and is negatively correlated with positive symptom severity [37]. Increased functional connectivity between the right middle temporal gyrus and left cerebellum is positively correlated with negative symptom severity [37]. Individuals at high risk of schizophrenia show intermediate changes in functional connectivity compared to controls and individuals with early illness schizophrenia [36, 37]. Hypoconnectivity of cognitive regions is seen in patients with schizophrenia, schizophreniform disorder and schizoaffective disorder. Conversely hyperconnectivity of somatomotor networks is noted, in keeping with neurological soft signs and altered action-perception found in these disorders. An altered sense of self-agency is putatively attributed to the reduced modularity of default mode and somatosensory networks [38].

Diffusion-weighted imaging (DWI) uses the magnetic resonance properties of water molecules as they diffuse along the axonal length of white matter fibers to trace, reconstruct and quantify these fibers in vivo [39]. As white matter bundles, the cerebellar peduncles may be identified using this technique. Altered white matter integrity has been found in individuals with schizophrenia in the left SCP compared to controls [40]. Neural disorganization in the left and right SCP has been associated with cognitive abnormalities in patients with schizophrenia [41] with MCP fractional anisotropy (FA), a proxy measure of axonal integrity, correlated to the dosage of neuroleptics required in schizophrenia [42].

As outlined, recent studies have indicated a possible ‘redefinition’ of the psychotic phenotype where, rather than as a distinct entity, psychosis may be viewed along a continuum. Early along such a continuum includes PEs that do not meet the full diagnostic criteria for a diagnosable psychiatric condition. Altered cerebellum connectivity and cerebellar peduncle diffusivity changes (in particular along the SCP) have been implicated in psychotic conditions (such as schizophrenia). However, no research to date has investigated the potential relationship between PE and cerebellar peduncles early along the psychosis spectrum (i.e., subclinical PEs). The current study seeks to fill this gap in the literature using high-resolution DWI to explore differences in cerebellar peduncles diffusion between adolescents with PEs and controls.

## Materials and Methods

### Participants

Participants were recruited as part of the Adolescent Brain Development Study [43]. Adolescents were recruited from 16 schools in counties Dublin and Kildare, Ireland. The age of recruitment was between 11 and 13. Full informed consent was obtained from the parents and the adolescents prior to the study commencing. The Strengths and Difficulties Questionnaire (SDQ) was used to identify psychopathology in participants. This validated instrument uses a questionnaire and computerized algorithms to predict opposition disorders, hyperactivity-inattention disorders, and anxiety-depressive disorders [44]. PEs were identified using the validated 7-item Adolescent Psychotic Symptoms Screener (APSS) [43]. Adolescents and their parents underwent a semi-structured diagnostic interview, the Schedule for Affective Disorders and Schizophrenia for School-aged Children, Present and Lifetime Versions (K-SADS-PL) [45]. Questions covering the five positive symptom sections of the Structured Interview for Psychosis – risk Syndromes (SIPS) (P1-P5), necessary to diagnose prodromal risk syndromes, and questions regarding onset, frequency, attributions for and distress caused by symptoms were added. For further details on the recruitment and interview assessments, see Kelleher et al. [2, 46]. All adolescents that reported any previous PEs and a random matched selection of adolescents reporting no PEs (controls) were invited for an MRI scan (see below). Exclusion criteria for both the PEs and control groups included contraindications for MRI, chronic neurological or medical illnesses, any long-term medications, and a history of a diagnosable psychiatric or developmental disorder.

Ethical approval was granted by the Beaumont Hospital Medical Ethics Committee.

### Diffusion-Weighted Imaging

MR data was acquired on a Philips (Best, Netherlands) Intera Achieva 3.0 Tesla MR system at Trinity College Institute of Neuroscience, Dublin. One hundred and eighty axial T1-weighted images (T1W-IR1150 sequence, TE = 3.8 ms, TR = 8.4 ms, field of view (FOV) 230 mm, 0.898 × 0.898 mm^2^, in-plane resolution, slice thickness 0.9 mm, flip angle alpha = 8°) were acquired. Whole brain, high angular diffusion imaging (HARDI) [47, 48] was acquired using a spin-echo echo-planar imaging pulse sequence (TE = 52 ms, TR = 11,260 ms, flip angle alpha = 90°), FOV 224 mm, 60 axial slices, 2 mm^3^ isotropic voxels, b-value = 1500 s mm^−2^ in 61 non-collinear gradient directions. A b0 image was also acquired.

### DWI Pre-processing and Whole Brain Tractography

*ExploreDTI* [49], a MATLAB [50] based diffusion magnetic imaging toolbox, was used for complete pre-processing, modelling and computation of diffusion data. All imaging data was anonymized and randomized from PE and controls into a single group prior to processing to reduce bias. Preprocessing, prior to tractography, included the following steps in sequential order: signal drift correction via linear and quadratic correction [51]; Gibbs artefact correction [52]; orientation checks via glyph inspection; head motion and eddy current correction using rigid body and affine registration respectively to b0 image [53]; and echo planar deformation corrections via affine registration to the T1 image [54]. These operations were performed using the latest version of toolbox plugins directly within *ExploreDTI*. All potential deterministic streamlines throughout the entire brain (i.e., whole brain tractography) were generated using constrained spherical deconvolution (CSD) modelling (as a recursive calibration of the response function) within *ExploreDTI* [55]. Whole brain seeding was used with seed voxel sizes of 2 mm 3 and a fiber orientation distribution threshold of 0.1. Whole brain tractography was achieved through multiple random seed placements of one seed per voxel [47, 56]. The step size was set at 0.5 mm and the maximum angle threshold was set at 89° to maximise all potential streamlines entering and exiting the peduncles. All whole brain streamline lengths were set between 10 and 500 mm [57]. The higher thresholds and lengths were chosen to capture as faithfully as possible the multiple fibers entering in and out of the peduncles and complex ascending and descending tracts of the brainstem.

### Isolation of Peduncles from Whole Brain Tractography

**Fig. 1:**
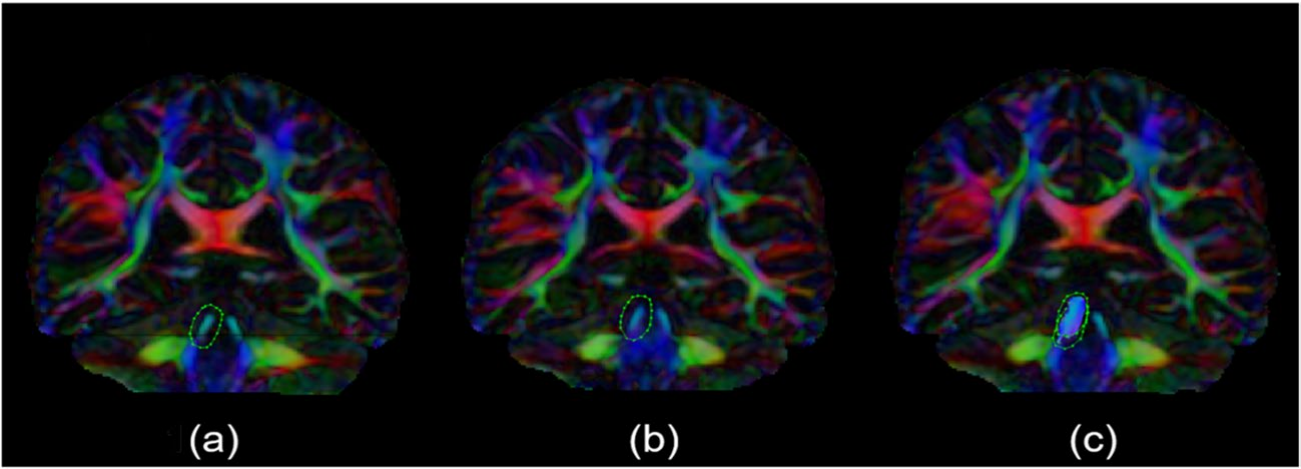
Superior Cerebellar Peduncle method. (**a**), (**b**) and (**c**) are FEFA (First Eigen-vector Fractional Anisotropy) images in the coronal plane at the level of the splenium of the corpus callosum, showing the sequential isolation of the right superior cerebellar peduncle

**Fig. 2:**
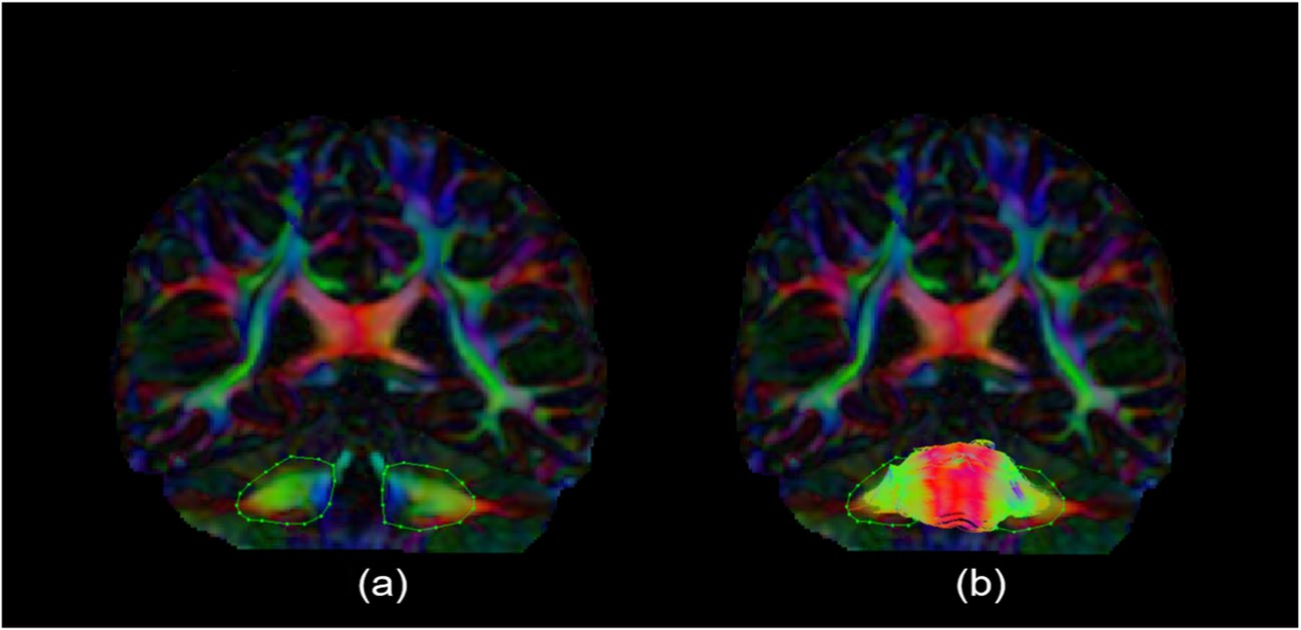
Middle Cerebellar Peduncle method. (**a**) and (**b**) are FEFA (First Eigen-vector Fractional Anisotropy) images in the coronal plane at the level of the splenium of the corpus callosum, showing the sequential isolation of the combined middle cerebellar peduncles

**Fig. 3:**
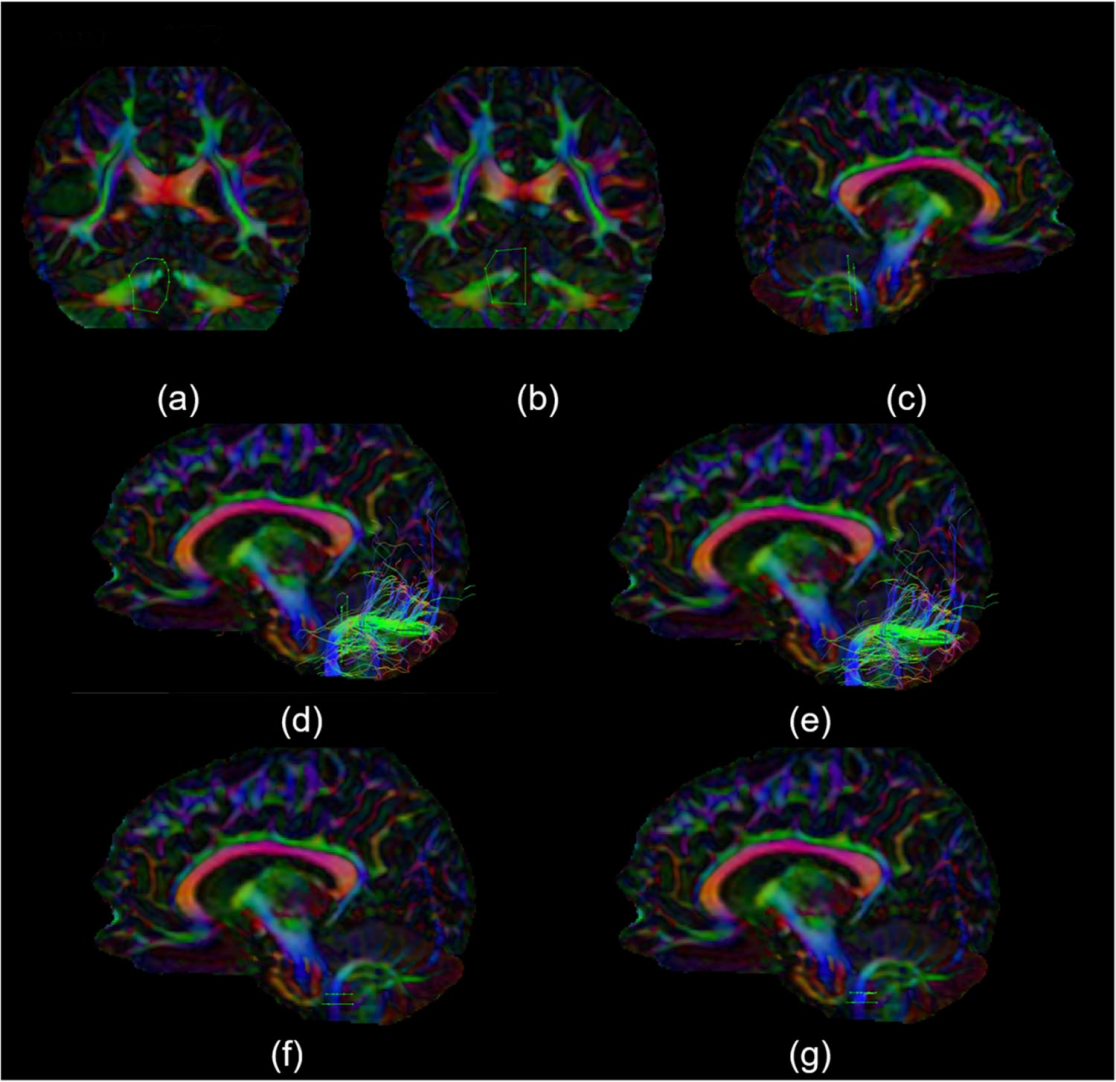
Inferior Cerebellar Peduncle method. (**a**) and (**b**) are FEFA (First Eigen-vector Fractional Anisotropy) images in the coronal plane at the level of the most posterior point of the splenium of the corpus callosum, and (**c**) to (**g**) are FEFA images in the sagittal plane to the left of the midline, showing the sequential isolation of the left inferior cerebellar peduncle

The superior and inferior (left and right) cerebellar peduncles and a single conjoined middle cerebellar peduncle were isolated individually using ‘AND’ and ‘NOT’ gates in *ExploreDTI* around anatomically determined landmarks. This bespoke protocol was generated following consultation with neuroanatomists familiar with the complex anatomy of the region at the Royal College of Surgeons, Dublin. Two raters were trained to isolate each peduncle in conjunction with the neuroanatomist. Each output was checked, examined and cleaned for extraneous streamlines inconsistent with known anatomy of the peduncle by the rater and the neuroanatomist. A comparison of reliability between each independent rater was assessed using interclass correlation coefficient (ICC) analysis for macroscopic measures (peduncle length and volume) and microscopic metrics (FA, MD, AD and RD). An ICC > 0.9 was considered sufficient for interrater reliability with the neuroanatomist and two raters working collaboratively to adjust output accordingly if needed. A second neuroanatomist resolved disputes if required and also assessed all peduncles for face validity. The peduncles were reconstructed with excellent inter-rater reliability for macroscopic and microscopic metrics. The following protocol (Figs. 1, 2, and 3) is based on the color coding in ExploreDTI using a FEFA (first eigenvector fractional anisotropy) map (i.e., the first eigenvector scaled by the FA map).

### Superior Cerebellar Peduncles

An AND gate was placed around the SCP where it is most separated from the MCP on the coronal plane (Fig. 1(a)). An AND gate was placed around the SCP immediately before fusion with the contralateral SCP on the coronal plane (Fig. 1(b)). Extraneous streamlines such as those of the MCP and brainstem were removed (cleaning) and the SCP was generated from segmentation using the original gates (Fig. 1(c)). This was repeated on the contralateral side.

### Middle Cerebellar Peduncles

The left and right Middle Cerebellar Peduncles (MCP) for our isolation purposes were treated as a single bundle. An AND gate was placed around the widest point of the MCP on the coronal plane on the left and right sides (Fig. 2(a)). This was immediately segmented and cleaned to give the final MCP segment (Fig. 2(b)).

### Inferior Cerebellar Peduncle

A ventral AND gate was placed around the Inferior Cerebellar Peduncles (ICP) where the pink fibers below are most visible (Fig. 3(a), (c)). A second AND gate was placed dorsal to this where orange is visible at the top of the ICP in the coronal plane (Fig. 3(b), (c)). This was then cleaned to remove the MCP and SCP (Fig. 3(d)). The original AND gates were removed. An AND gate was drawn around the ICP in the axial plane below the curve as seen on sagittal views, and another around the ICP 5 slices caudal to this (Fig. 3(e), (f)). These 2 AND gates were used to segment the final ICP for statistical analysis (Fig. 3(g)).

### Statistical Analysis

All statistical analyses were performed on IBM SPSS Statistics 26. Outliers were defined as being either the 3rd quartile + 1.5*interquartile range or the 1st quartile – 1.5*interquartile range. These outliers were systematically removed as part of data pre-processing. One-way between-group analyses of covariance (ANCOVA) were used to ascertain the mean differences between the PE and control groups in the macroscopic and microscopic measures of all generated bundles. Age, sex, and estimated total intracranial volume (eTIV) were controlled as covariates. Effect sizes were calculated as partial eta^2^ (with the interpretation that 0.01 = low, 0.09 = moderate and 0.25 = large). Bonferroni corrections were performed for multiple comparisons throughout (5 discrete peduncles, 1 macroscopic metric [volume] and 4 microscopic metrics [FA, MD, RD, AD] in each peduncle = 0.05/25 = 0.002). The Bonferroni adjusted *p*-value threshold was 0.002 (based on multiple comparisons throughout the sample, with an α = 0.05).

## Results

### Demographics

Sixteen participants with PE (14 female) and seventeen healthy controls (7 female) were included in the final analysis (total = 33). Participants with PEs had at least one documented PE in the past, with the range being between 1 and 5 on the APSS. The mean APSS score was 2.2, suggesting a low level psychotic experience, with most (13 participants) reporting PEs within the previous 3–6 months. None of the PE or control group had any first-degree relatives with a diagnosed psychotic disorder. The mean age was 12.5 years. The PE group had significantly more females than males (χ2 = 7.643; *p* = 0.006); however, there were no differences between the PE and controls groups for handedness or age (age: *F* = 0.674; *p* = 0.418).

### Cerebellar Peduncle Metrics

No significant macroscopic or microscopic metric differences were found between young adolescents with PEs and controls for the SCP, MCP and ICP (Table 1). The SCP exhibited a trend towards reduced FA in the PE group (right; *p* = 0.045), (left; *p* = 0.058) but this did not survive correction for multiple comparisons.

**Table 1:** Cerebellar peduncle between group differences

	SCP		MCP	ICP	
	Left	Right		Left	Right
Volume	0.987	0.139	0.598	0.446	0.152
FA	*0.058*	0.045*	0.585	0.746	0.963
MD	0.797	0.379	0.417	0.624	0.552
AD	0.289	0.92	0.62	0.909	0.448
RD	0.692	0.166	0.392	0.592	0.691

ANCOVAs showing differences between young adolescents with PEs and controls for volumes and microscopic diffusion metrics of the ICP, MCP and SCP. Arrows show the direction of difference between PEs and controls. All analyses were corrected for age, sex and eTIV. No results reached the Bonferroni corrected significance threshold (*p* = 0.002). However, FA was lower at a trend level for the left (italics) and right (bold) SCP. AD, axial diffusivity; ANCOVA, analysis of covariance; eTIV; estimated total intracranial volume; FA, fractional anisotropy; ICP, inferior cerebellar peduncle; MD, mean diffusivity; MCP, middle cerebellar peduncle; RD, radial diffusivity; SCP, superior cerebellar peduncle (Table 2).

**Table 2:** Descriptive measures of peduncle data. Means and standard deviations of volumes and diffusion metrics in young adolescents with PEs and controls

	Group	Mean	Std. deviation
Left inferior cerebellar peduncle			
Approximate volume (mm3)	Control	184.1796912	43.00091078
	PE	165.3857869	42.81450029
Mean Fractional Anisotropy	Control	0.549750414	0.088899428
	PE	0.521553795	0.108808539
Mean Diffusivity	Control	0.000735247	8.26E-05
	PE	0.000756593	9.49E-05
Axial Diffusivity	Control	0.001236355	6.55E-05
	PE	0.001226693	8.39E-05
Radial Diffusivity	Control	0.000484691	0.000111868
	PE	0.00052154	0.000132444
Right inferior cerebellar peduncle			
Approximate volume (mm3)	Control	167.4984573	32.58100624
	PE	177.1469859	26.65161803
Mean Fractional Anisotropy	Control	0.568880971	0.08951802
	PE	0.551666453	0.090147727
Mean Diffusivity	Control	0.000722866	7.13E-05
	PE	0.000721517	8.40E-05
Axial Diffusivity	Control	0.001244381	8.03E-05
	PE	0.001215005	8.64E-05
Radial Diffusivity	Control	0.000462107	0.000101141
	PE	0.000474776	0.000111321
Left superior cerebellar peduncle			
Approximate volume (mm3)	Control	343.5733845	128.433044
	PE	368.3727832	108.9083684
Mean Fractional Anisotropy	Control	0.514863227	0.040769661
	PE	0.552209935	0.055738156
Mean Diffusivity	Control	0.001085619	0.000151587
	PE	0.001103596	0.000109486
Axial Diffusivity	Control	0.001753158	0.000213914
	PE	0.001844213	0.00013838
Radial Diffusivity	Control	0.000751852	0.000129923
	PE	0.000733286	0.000115758
Right superior cerebellar peduncle			
Approximate volume (mm3)	Control	324.4049742	83.38330209
	PE	350.8767846	100.5867483
Mean Fractional Anisotropy	Control	0.513994223	0.037242792
	PE	0.555622734	0.050365458
Mean Diffusivity	Control	0.001107803	0.000124752
	PE	0.001052754	9.91E-05
Axial Diffusivity	Control	0.001787375	0.000176395
	PE	0.001769306	0.000146703
Radial Diffusivity	Control	0.000768018	0.000110537
	PE	0.000694476	9.81E-05
Middle cerebellar peduncle			
Approximate volume (mm3)	Control	10,087.38661	1443.439439
	PE	9887.972964	1581.559026
Mean Fractional Anisotropy	Control	0.518415295	0.024681231
	PE	0.528308651	0.02937006
Mean Diffusivity	Control	0.000863248	5.20E-05
	PE	0.000834783	4.89E-05
Axial Diffusivity	Control	0.001399126	5.61E-05
	PE	0.00137107	6.24E-05
Radial Diffusivity	Control	0.000595305	5.37E-05
	PE	0.000566638	5.12E-05

## Discussion

The results of this pilot study provide additional perspective on potential mechanisms of cognitive dysmetria in young adolescents at the early end of the psychotic spectrum but not reaching a threshold for diagnosis. Specifically, a trend towards reduced FA in the right (*p* = 0.045) and left (*p* = 0.058) SCP was found in the PE group.

White matter anomalies have been found in many psychiatric disorders using DWI function [58, 59]. Upon investigation of patients with first-episode psychosis, a study of 116 identified 4 clusters of white matter fibers with significantly reduced FA when compared to controls, these include the superior and inferior longitudinal fasciculus bilaterally, forceps major, anterior and superior thalamic radiation and corpus callosum [60]. The observed white matter deficits indicate suggestive involvement of interhemispheric connections, corticosubcortical pathways, and fronto-temporal and fronto-occipital connections in early course schizophrenia in concordance with previous studies [61-63]. Further studies have shown FA deficits specifically in adolescents with early-onset psychotic symptoms also finding reductions in the frontal and occipital white matter [64]. Studies have also implicated deficits in the hippocampus and corresponding output fibers, the fornix, in young adults with sub-threshold psychotic symptoms [65, 66]. Cingulum white matter changes have also been found in non-clinical young adolescents with psychotic experiences [67]. Despite evidence of FA changes in early psychosis, not all studies show these changes [68-70]. One cross-sectional study demonstrated widespread reduced FA values in regions including frontal white matter, the corpus callosum, and the frontal longitudinal fasciculus patients with chronic schizophrenia [68]. However, patients with first-episode psychosis showed only trend level reductions in FA, suggesting that first-episode patients may exhibit more subtle diffusion changes compared to more established changes further along the spectrum in chronic schizophrenia.

Studies investigating the diffusion of the cerebellar peduncles in persons with psychosis, or risk for psychosis, have uncovered altered white matter integrity of the SCP [40, 71, 72] and MCPs [42] as compared to healthy controls. Our study fails to replicate such findings and may be attributable to several factors. Firstly, most of these past studies investigate functional connectivity in adult persons diagnosed with a serious psychotic illness, such as schizophrenia [38, 73]. Our cohort of participants differs both in terms of age and level of psychosis, that is, participants had not been previously diagnosed with a psychotic disorder but were rather recruited from the general school going population [43]. As with our findings, the one study which investigated FA in adolescents with first-admission schizophrenia also found no peduncle differences compared to healthy controls [74]. Notwithstanding, adolescents at ultra-high-risk for psychosis have been shown to progressively exhibit reductions in FA in the SCPs over a 12-month period while healthy controls show normative increases [75]. The SCP is the main efferent from the cerebellum to the thalamus and cerebral cortex linking a wide range of cognitive functions. Disruption of this connectivity could account for changes in thinking and perception found in PEs. Our PE group is a considerably more subtle phenotype than ultra-high-risk or first episode psychosis, being the closest phenotype to controls along the spectrum (i.e., have never presented to a clinician for symptoms). However, it is intriguing that even in our cohort with subtle symptoms, SCP changes can be uncovered in young adolescents with PE even if they don’t reach the threshold for a formal diagnosis. This may suggest that SCP changes start occurring even earlier along the psychosis spectrum than previously thought.

Subtle neurological abnormalities in sensory and motor performance known as Neurological Soft Signs (NSS) are also evident in persons with psychosis [76] and high-risk individuals [77-79]. Notably, ultra-high-risk adolescents have exhibited NSS across domains that predict longitudinal decreases in cerebellar-thalamic FA [75], and cerebellar soft signs (CSS) specifically have been noted in schizophrenic and bipolar disorder patients [80]. The presence of these abnormalities in children and adolescents suggests that such neurological dysfunction is reflective of an abnormal neurodevelopmental trajectory that offers a potential and viable biomarker for understanding the pathogenic progression of psychosis during the adolescent risk period. The cortico-cerebellar-thalamic-cortical circuitry, specifically at the level of the cerebellar peduncles warrants more attention regarding its contribution to psychosis, particularly in adolescent populations where the early stages of these disorders are detectable.

Diffusion tensor imaging is a non-invasive tool that can be used to indirectly reveal neuronal integrity reflecting neuronal organization and myelination [81]. This is the first study to investigate the diffusion properties of the cerebellar peduncles in young adolescents on the pre-diagnostic psychotic spectrum. This study used a bespoke peduncle isolation protocol that was informed by neuroanatomists and isolated by two independent raters with excellent inter-rater reliability. A further strength of this study is the inclusion of general school-going adolescents before they present to medical services, representing that earliest progression along the psychosis spectrum. Our trend level changes in the SCP in young people with PEs suggests that the SCP may have use as vulnerability marker following a larger replication study.

Although these findings suggest that the structural integrity of white matter in these participants was not disrupted, this study is limited by its small sample size. A larger sample may unearth more significant findings from our trend level superior peduncle findings and we would recommend replication of this study. Although we controlled for sex, the predominance of females in the PE group (14 females out of 16 participants) may also influence our null findings as females are known to generally have a different time course (later onset) and symptom profile (more affective symptoms rather than negative psychotic symptoms) than males [82]. As our study recruited young adolescents from the general school-going population, rather than a clinical high-risk population, and these PEs did not cause the participants to present to the medical system, clear unambiguous structural changes in highly heterogeneous pathways such as the cerebellar peduncles are unlikely in this very subtle phenotype.

Deterministic tractography is an experimental method subject to some technical and interpretation challenges (Jones, 2008). Known issues involve misinterpreting curving and kissing fibers. We used a high angle (89°) and small step-size (0.5 mm) for whole brain tractography to capture as many streamlines as possible from which we carved out our peduncles. The peduncles lie adjacent to and connect into the busy brainstem with its many ascending and descending fibers. CSD was chosen as the most appropriate tractography technique due to its potentially superior ability at detecting complex crossing and kissing fibers in a recent analysis of fiber estimation approaches (Wilkins et al., 2015).

As with all diffusion-weighted reconstruction, the generated peduncles in our study only describes the diffusivity in these regions of interest and it is assumed that this corresponds to actual anatomical white matter structures. An experienced neuroanatomist with expertise of the region aided the raters during cleanup. We felt this represented the most valid approach for removing extraneous streamlines. As the protocol was designed to consistently encompass as much of the peduncles as possible, the initial gates were overly broad. However, this resulted in a large amount of cleanup of extraneous streamlines. The average time of cleanup (both rater and neuroanatomist working together) was estimated at about 20 min per peduncle, per subject. It is appreciated that this approach may not be available or practical for some centers. However, with adequate training, this limitation may be overcome and a researcher/technician experienced with ExploreDTI and the complex regional anatomy could single-handedly undertake the entire process.

## Conclusion

This pilot study investigated whether differences in cerebellar peduncle integrity exist in a non-clinical sample young adolescents with psychotic experiences compared to controls using high-resolution diffusion-weighted imaging. Promising trend level reductions in FA (*p* = 0.045 and 0.058) were found in the right and left cerebellar peduncles. These reflect similar but more profound changes in the superior cerebellar peduncle in ultra-high-risk individuals and those with psychotic disorders. Superior cerebellar peduncle circuitry perturbations may occur early along in the psychosis spectrum and may potentially be a vulnerability marker for development of clinical psychosis.

## Data Availability

Data and materials are available by contacting the corresponding author.
